# Intraoperative Administration of Dexmedetomidine and Dexamethasone in Local Anesthetic Infiltration to Improve Postoperative Pain Control After Posterior Cervical Fusion

**DOI:** 10.7759/cureus.14699

**Published:** 2021-04-26

**Authors:** Warren A Southerland, Justin Gillis, Ivan Urits, Alan D Kaye, Jonathan Eskander

**Affiliations:** 1 Department of Anesthesia, Critical Care, and Pain Medicine, Beth Israel Deaconess Medical Center, Harvard Medical School, Boston, USA; 2 Department of Anesthesiology, Louisiana State University Shreveport, Shreveport, USA; 3 Anesthesiology and Pain Medicine, Portsmouth Anesthesia Associates, Portsmouth, USA

**Keywords:** dexmedetomidine, dexamethasone, dex-dex, regional anesthesia, peripheral nerve blocks, spine surgery, postoperative pain, pain control, multimodal analgesia, cervical fusion

## Abstract

Dexmedetomidine, a selective and potent α2-adrenoceptor agonist, is used for its anxiolytic, sedative, and analgesic properties. Dexamethasone is a high-potency, long-acting glucocorticoid that has been shown to provide analgesic and anti-inflammatory effects. At present, little has been published with regard to the effectiveness of these drugs as dual agents with local anesthetics for analgesia. In this report, a case of a 50-year-old man who underwent a cervical spine orthopedic procedure is described, in which an intraoperative injection of ropivacaine was administered with the adjuvants dexmedetomidine and dexamethasone, providing extended postoperative pain relief. In summary, we present a patient who had an injection of ropivacaine with dexmedetomidine and dexamethasone into the erector spinae muscles in the cervical region, which provided improvement in postoperative pain and reduced opioid consumption for five days post-surgery, demonstrating additive and/or synergistic effects beyond the normal local anesthetic duration.

## Introduction

Dexmedetomidine, a selective and potent α2-adrenoceptor agonist, is clinically used for its anxiolytic, sedative, and analgesic properties [1]. Compared to clonidine, another α2-agonist that has been in use for decades, it has greater selectivity for the α2-receptors and, as such, is a more potent sedative than clonidine. Dexmedetomidine has other features that make it an attractive alternative sedative choice as well, including minimal influence on respiration, and when administered, patients remain easily arousable. The main adverse effects of dexmedetomidine are alterations in hemodynamic status, which can include hypertension, bradycardia, and hypotension via pre-and postsynaptic α2-receptor activation. This can lead to vasoconstriction, vasodilatation, and reflex bradycardia [2]. Originally, it was approved for intravenous (IV) administration for sedation of patients who were mechanically ventilated in the intensive care unit. It could be administered for up to 24 hours. In 2008, indications for the use of dexmedetomidine were expanded for the sedation of non-intubated patients periprocedurally [2]. In addition, dexmedetomidine has been used in regional anesthesia as an adjunct to local anesthetics to prolong the density and duration of peripheral nerve blocks [3,4].

Dexamethasone is a high-potency, long-acting glucocorticoid, which has been demonstrated to provide analgesic and anti-inflammatory effects [5]. Its analgesic mechanism of action is not completely understood, but it may arise from decreased nociceptive C-fiber activity from a direct effect on the glucocorticoid receptor [6], vasoconstrictive effects leading to reduced local anesthetic vascular uptake [7], and/or delivering systemic anti-inflammatory effects [8]. Dexamethasone has many clinical uses, including its use in interventional pain procedures to reduce swelling and/or inflammation of nerves as well as being an adjunct to local anesthetics for peripheral nerve blocks [3-5,9].

In this report, we present a case of a 50-year-old man who underwent a posterior cervical fusion with intraoperative wound infiltration using 20 mL of 0.2% ropivacaine plus 25 mcg dexmedetomidine and 5 mg preservative-free dexamethasone for improved postoperative analgesia.

## Case presentation

A 50-year-old Caucasian male with a history of hypertension, diabetes mellitus type II, previous hemorrhagic stroke, and cervical spondylitis with concomitant stenosis complicated by myelopathy was admitted for surgery. The patient presented for a laminectomy and fusion at cervical levels C3 through C7. Prior to the procedure, anesthesia and surgical informed consents were obtained from the patient by the respective teams. Intraoperatively, 20 mL of ropivacaine with 5 mg of dexamethasone and 25 mg of dexmedetomidine was injected both superior to and inferior to the erector spinae muscles at cervical levels C3, C5, and C7 bilaterally.

The patient’s self-described pain level in the post-anesthesia care unit was a 3/10 using the Numeric Rating Scale (NRS-11). Over the first two postoperative days, the patient described his pain as 5/10, and he required 10 mg of oxycodone twice daily for breakthrough pain. In terms of sensation, the patient described numbness along the surgical incision along with self-described feelings of muscle cramping at the surgical site through postoperative day 5. Of note, throughout the patient’s postoperative course, he was able to sleep overnight ranging from 6 to 8 hours without requiring any doses of Roxicodone®. He described a pain level of 8/10 starting on postoperative day 5, and his dose of Roxicodone was increased to 10 mg four times daily to control his pain. The surgical block had worn off by postoperative day 5. After an increase in opioid consumption on postoperative day 5, he was able to quickly wean off shortly thereafter.

## Discussion

In our patient, the injection of dexmedetomidine and dexamethasone (Dex-Dex) into the erector spinae muscles in the cervical region provided improvement in postoperative pain. Previous studies have examined dexmedetomidine’s use as an adjunct to peripheral nerve blocks with increased post-procedure pain control and an increase in time to first request for additional pain medication [10,11]. A study by Obayah et al. showed an increase in the time to first analgesic request following greater palatine nerve blocks cleft palate repair in children when bupivacaine plus dexmedetomidine (1 µg/kg) was compared with bupivacaine alone (22 hours vs. 14.2 hours; p < 0.001) [10]. In addition, pain scores in the dexmedetomidine group were significantly lower for the first 24 hours [3]. A systematic review and meta-analysis by Abdallah et al. found that when IV dexmedetomidine accompanied spinal anesthesia, sensory block duration was prolonged by at least 34% (point estimate: 38%%), p<0.01, and time to first analgesic request was increased by at least 53% (point estimate: 60%), p<0.01 [11]. The addition of dexmedetomidine to local anesthetic is an area of pain medicine that has continued to provide promising results.

Dexamethasone has shown promise as an adjunct as well. A study by Schnepper et al. showed that increased block duration was associated with receiving any dose of perineural dexamethasone (p < 0.01) [12]. Multiple studies have also shown that perineural dexamethasone is superior to systemic administration for prolonged analgesia [9,13].

Postoperative pain control is an issue with orthopedic surgery, including spine surgery [14,15]. Multimodal analgesia has been the preferred choice over opioids in recent years, including regional anesthesia with peripheral nerve blocks. This case shows how an intraoperative wound infiltration with Dex-Dex can help with postoperative pain.

The use of both dexamethasone and dexmedetomidine together has been theorized to prolong the duration of local anesthetics and provide longer analgesia, as seen in our described patient [16]. The actual mechanism by which the drugs interact to produce this effect is not known but may arise from multiple factors. For example, vasoconstriction caused by both dexamethasone and dexmedetomidine maintains the concentration of local anesthetic around the targeted nerve and inhibits the nociceptive signal transmission by myelinated C fibers [16]. Dexmedetomidine may also inhibit hyperpolarization-activated cation current, stimulate the release of enkephalin-like substances at peripheral sites, and block the signals through C and Aδ fibers [16]. Due to the various and different ways the two agents can prolong the duration of action of local anesthetics and analgesia, a synergistic effect can arise from an additive effect from these actions.

The efficacy of either agent as adjuncts in peripheral nerve blocks has been compared [17]. Longer sensorimotor block and analgesia were demonstrated by dexamethasone.

As for their potential synergistic effect, Zhang et al. found that the addition of combined perineural dexmedetomidine and dexamethasone to ropivacaine for intercostal nerve blocks provided prolonged analgesia for patients undergoing thoracoscopic pneumonectomies [16].

Additionally, the study monitored for potential side effects from the two-drug combination, including hypotension, hypoxemia, respiratory depression, vomiting, nausea, pruritus, and dizziness [16]. No significant differences were observed in the incidences of side effects among the four study groups (intercostal nerve block with ropivacaine only, ropivacaine and dexamethasone, ropivacaine and dexmedetomidine, and ropivacaine/dexamethasone/dexmedetomidine) [16]. Recently, some authors demonstrated that the Dex-Dex combination could provide several days of postoperative pain relief from perineural administration [18-20]. Continued research into dexmedetomidine with dexamethasone as an intraoperative infiltrate is warranted.

## Conclusions

Dex-Dex has been shown to be effective adjuncts to local anesthetics in peripheral nerve blocks. However, their efficacy together has not been thoroughly researched. As shown in our case, the use of both agents together as an intraoperative injection improved postoperative pain control and reduced opioid consumption in a patient undergoing cervical spine surgery. These findings point towards a possible additive and/or synergistic effect, which can potentially help improve postoperative pain control for spine surgery patients and potentially increase the efficacy of other peripheral nerve blocks. Further studies are needed to confirm the potential benefits of the combination of Dex-Dex extending the analgesic duration when combined with local anesthetics.
